# From molecular mechanisms to nutritional applications: protein and polyphenol interventions in sarcopenia

**DOI:** 10.1007/s13105-026-01154-6

**Published:** 2026-02-16

**Authors:** Patricia Aragón-Espinosa, Patricia Pérez-Matute, Catherine Bompart, Marine Gueugneau, Frederic Capel, Jose M. Arbones-Mainar, Saioa Gomez-Zorita, Anna Mas-Capdevila, Xavier Escoté, Montserrat Pinent, Anna Ardévol

**Affiliations:** 1 Eurecat, Technological Unit of Nutrition and Health, Centre Tecnològic de Catalunya, Avinguda Universitat 1, Reus, Catalonia 43204 Spain; 2 Universitat Rovira I Virgili ( Department Biochemistry and Biotechnology), Reus, 43201 Spain; 3 Institute of Health Pere Virgili (IISPV), Reus, 43204 Spain; 4 CIBER in Physiopathology of Obesity and Nutrition (CIBEROBN), Carlos III Health Institute, Madrid, 28029 Spain; 5 Center of Environmental, Food and Toxicological Technology-TecnATox, Rovira i Virgili University, Reus, 43201 Spain; 6 Universitat Rovira i Virgili (Department of Biochemistry and Biotechnology), Tarragona, 43007, Spain; 7 Unité de Nutrition Humaine, Université Clermont Auvergne, INRAE, Clermont-Ferrand, 63000 France; 8 Lifestyle, Microbiota and Health Group. Health Sciences Faculty, University of La Rioja, Logroño, La Rioja 26003 Spain; 9 Adipocyte and Fat Biology Laboratory (AdipoFat), Instituto Aragonés de Ciencias de la Salud (IACS), Instituto de Investigación Sanitaria Aragón, Zaragoza, 50009 Spain; 10 Nutrition and Obesity Group, Department of Nutrition and Food Science, University of the Basque Country (UPV/EHU) and Lucio Lascaray Research Institute, Vitoria-Gasteiz, 01006 Spain; 11 Bioaraba Health Research Institute, Vitoria-Gasteiz, 01006 Spain

**Keywords:** Sarcopenia, Muscle, Ageing, Protein, Polyphenol

## Abstract

Sarcopenia, the progressive loss of skeletal muscle mass and function, represents a major public health concern due to its impact on mobility, independence, and quality of life, especially in oldadults. Current treatment strategies primarily rely on resistance training and nutritional support, with particular emphasis on adequate protein intake to stimulate muscle protein synthesis. In this review, we provide an overview of the pathophysiology of sarcopenia, with a focus on the molecular mechanisms underlying muscle atrophy and dysfunction. We explore the role of dietary protein as a cornerstone of sarcopenia management, highlighting current evidence on optimal protein sources and intake strategies. In addition, we examine other nutritional interventions, placing special emphasis on polyphenols, naturally occurring compounds known for their antioxidant and anti-inflammatory properties, which have shown promise in modulating pathways relevant to muscle preservation. Vitamin D and other nutritional supplements are also discussed in the context of their potential to support muscle health. Finally, we address emerging trends in the field, including targeting microbiota. By integrating current findings, this narrative review aims to provide a compilation of the evidence-based nutritional interventions for the prevention and management of sarcopenia.

## Introduction

Sarcopenia is “a progressive and generalised skeletal muscle disorder involving the accelerated loss of skeletal muscle mass and function“ [1], primarily affecting ageing populations.It is recognised as a distinct diagnosis in the International Classification of Diseases, 10th Revision, Clinical Modification (ICD-10-CM) since 2016 [2]. Sarcopenia is classified as primary when age-related decline is the sole cause, and secondary when associated with underlying diseases or conditions [3]. There is no consensus regarding the relative prevalence of both types because it depends on several factors, and most studies do not differentiate between them [1, 2]. The prevalence of sarcopenia varies depending on the population studied, the diagnostic criteria used, and the geographical location [1, 2]. Thus, accurately describing its distribution is challenging due to variations in operational definitions, which result in widely differing prevalence estimates. Prevalence tends to be higher when sarcopenia is defined solely by low [1–3]lean mass compared to definitions incorporating muscle strength and physical function [1–3]. An earlier review pooling data from 58 study populations across 26 countries found prevalence estimates between 10% and 40%, depending on the definition used [4]. Even the most conservative estimates indicate that 5–10% of the general population is affected, with prevalence increasing significantly with age, particularly among older institutionalised populations. Longitudinal studies further highlight sarcopenia’s progression, with substantial proportions of older adults developing it over relatively short follow-up periods (e.g., 15% over 8 years in the English Longitudinal Study of Ageing) [5]. Sex differences in sarcopenia prevalence are inconsistent, varying with the definitions applied. For instance, European criteria show a higher prevalence in men, whereas American criteria suggest a higher prevalence in women, and Asian criteria reveal no sex difference [2]. Regional differences in prevalence have also been observed, but these are difficult to summarise due to limited data availability in certain regions (e.g., Africa) [3]. Global variations in sarcopenia’s components, such as grip strength, could also be responsible for these discrepancies [6].

Diagnosis typically involves identifying reduced muscle mass and strength below standardised thresholds in the absence of other causes. It is associated with an increased risk of falls and fractures, loss of independence in daily activities, higher rates of hospitalisation, surgical complications and elevated morbidity and mortality. Understanding its prevalence, aetiology, and impact is crucial for developing effective prevention and treatment strategies, particularly in the context of an increasingly ageing global population. Addressing sarcopenia is vital to mitigating its impact on public health, particularly in the context of an ageing global population [7, 8].

Sarcopenia is a multifactorial condition driven by age-related processes such as neurodegeneration, hormonal changes, chronic inflammation, mitochondrial dysfunction, inadequate protein intake, and physical inactivity. Nutritional interventions are particularly promising in mitigating these effects, with protein quality and availability being central to muscle structure and function. Beyond proteins, bioactive compounds, especially polyphenols, possess potent antioxidant and anti-inflammatory properties, yet their role in sarcopenia remains underexplored. This review summarises the key molecular mechanisms underlying sarcopenia. It also examines current evidence on the preventive and protective effects of polyphenols in combination with proteins. By linking these compounds to muscle structure and function, we highlight their potential as promising candidates for preventing age-related muscle decline.

To ensure a comprehensive and balanced overview of the current evidence, this review was developed in accordance with the SANRA (Scale for the Assessment of Narrative Review Articles) principles [9]. A structured literature search was conducted in *PubMed/MEDLINE*,* Scopus* and *Web of Knowledge* covering publications from January 2000 to February 2025. The search strategy combined key terms and MeSH headings, including “muscle atrophy”, “oxidative stress”, “inflammation”, “mitochondrial dysfunction”, “protein synthesis”, “IGF-1/Akt/mTOR”, “FOXO”, “NF-κB”, “MAPK”, “Wnt/β-catenin”, “sarcopenia” and “ageing”. To address the nutritional dimension, additional keywords such as “dietary protein”, “polyphenol”, “flavonoid”, “resveratrol”, “catechin”, *“*omega-3 fatty acids” OR “vitamin D” OR “probiotics” OR “exercise” OR “resistance training” OR “myostatin” OR “gut microbiota” and “synergy OR interaction OR complex OR conjugate” were incorporated. The initial search identified 1,200 Titles, and abstracts were screened for relevance, prioritising studies addressing human populations or translational research with direct implications for ageing muscle physiology. Animal and mechanistic studies were also included when there was a lack of human studies. This process yielded approximately 250 core references, from which the most representative and high-quality studies were integrated to support a balanced and evidence-based discussion of current and emerging therapeutic perspectives. Some additional references were retrieved through citation tracking, and AI-assisted language tools were used to support text refinement.

## Pathophysiology of sarcopenia

Due to the complexity of the aetiology of sarcopenia, understanding the intricate mechanisms underlying muscle atrophy, the roles of oxidative stress and inflammation, and the dysregulation of key molecular pathways is crucial for developing effective strategies to prevent and treat sarcopenia (summarised in Table 1).

**Table 1:** A summary of the pathophysiology of muscle sarcopenia and the protective effects of protein or polyphenols is described

Pathophysiology of Sarcopenia	Protein protective effects	Polyphenols protective effects
Less responsiveness of IGF-1/Akt/mTOR signalling pathway	[10–12]	[1, 13]
Increased FOXO activity	[14]	[15]
Activation of NF-κB		[16]
Inhibition of Wnt signalling		[17]
Dysregulation of MAPK signalling		[18]
Atrophy of type II fibres	[19]	[20]
Destabilisation of the NMJ and subsequent denervation of muscle fibres	[21]	[22]
Dysfunctional satellite cells	[23]	[24]
Alterations in ECM composition and structure	[25]	[26]
Inflammaging	[27]	[13]
Oxidative stress	[27]	[28]
Myosteatosis		
Mitochondrial integrity compromised		[29]
Disruptions in Ca^2+^ homeostasis	[30]	[31]
Dysregulation of miRNA expression		[32]
Loss of Proteostasis		[33]

Legends: IGF-1, insulin-like growth factor 1; Akt, protein kinase B; mTOR, mechanistic target of rapamycin; FOXO, forkhead box O transcription factors; NF-κB, nuclear factor kappa-light-chain-enhancer of activated B cells; Wnt, wingless/integrated signalling pathway; MAPK, mitogen-activated protein kinase; Type II fibres, fast-twitch skeletal muscle fibres; NMJ, neuromuscular junction; ECM, extracellular matrix

### Muscle atrophy mechanisms in sarcopenia

Muscle atrophy, characterised by reduced muscle mass and strength, is a key feature of sarcopenia. This complex process results from an imbalance between protein synthesis and degradation, along with disruptions in cellular function, neuromuscular integrity, and metabolic regulation. Next, we summarise the most clearly defined mechanisms:

#### Imbalance between protein synthesis and degradation

Muscle mass is maintained by a balance between anabolic (protein synthesis) and catabolic (protein degradation) processes. Ageing and various pathological conditions disrupt this balance, shifting it towards increased protein degradation and reduced protein synthesis, ultimately leading to muscle atrophy [33]. The IGF-1/Akt/mTOR signalling pathway, crucial for muscle protein synthesis, becomes less sensitive to stimuli like leucine intake and exercise in the elderly, impairing the diet-induced enhancement of protein synthesis [34, 35].

#### Changes in myofibers

Sarcopenic muscle exhibits a reduction in the size and number of myofibers, particularly affecting type II (fast-twitch) fibres. This preferential atrophy of type II fibres contributes to the decline in muscle strength and power observed in sarcopenia [36]. Additionally, intramuscular and intermuscular fat infiltration (myosteatosis) occurs, further compromising muscle quality and function [37].

#### Loss of proteostasis

Proteostasis, the maintenance of protein homeostasis, is crucial for proper cellular function. In sarcopenia, dysregulation of proteostasis leads to the accumulation of misfolded and aggregated proteins, contributing to myofiber atrophy and impaired muscle function [38]. Impaired autophagy in aging leads to protein aggregate build-up, which further exacerbates muscle degeneration [39].

#### Denervation

The neuromuscular junction (NMJ), the interface between motor neurons and muscle fibres, plays a critical role in muscle function. Aging-related changes in NMJ morphology and function lead to destabilization of the NMJ and subsequent denervation of muscle fibres. This loss of motor innervation contributes to muscle atrophy and functional decline [40].

#### Dysfunctional satellite cells

The decreased activity of these primary muscle stem cells limits the body’s ability to replace or repair damaged muscle fibres, furthering atrophy [41].

#### Mitochondrial dysfunction

Mitochondria, the powerhouses of the cell, are essential for energy production and muscle function. In sarcopenia, mitochondrial integrity is compromised, leading to reduced energy production, increased oxidative stress, and impaired mitochondrial dynamics. The accumulation of damaged and dysfunctional mitochondria further exacerbates muscle atrophy [42].

#### Ionic dyshomeostasis

Proper calcium (Ca^2+^) signalling is critical for muscle contraction and relaxation. Ageing-related disruptions in Ca^2+^ homeostasis impair muscle function and contribute to muscle atrophy [43].

#### Extracellular matrix dysfunction

Age-related remodelling of the extracellular matrix (ECM), the structural support system of muscle tissue, also plays a role in sarcopenia. Alterations in ECM composition and structure affect muscle fibre mechanics and contribute to muscle weakness and functional decline [44].

### Molecular pathways involved in muscle atrophy

Moving towards a molecular level, several signalling pathways are implicated in the regulation of muscle mass and are dysregulated in sarcopenia. These pathways include:

#### IGF-1/Akt/mTOR pathway

This pathway is a major regulator of protein synthesis and muscle hypertrophy. Activation of the IGF-1 receptor stimulates the phosphorylation of Akt, which in turn activates mTORC1, a key regulator of protein synthesis. In sarcopenia, the IGF-1/Akt/mTOR pathway becomes less responsive to anabolic stimuli, leading to reduced protein synthesis and muscle atrophy [34, 45].

#### FOXO transcription factors

FOXO transcription factors regulate the expression of genes involved in muscle protein degradation, autophagy, and antioxidant defence. During muscle atrophy, FOXO activity is increased, leading to the upregulation of genes that promote protein breakdown and inhibit muscle growth [46].

#### NF-κB signalling

This signalling pathway is a key mediator of inflammation. Activation of NF-κB leads to the production of pro-inflammatory cytokines, which promote muscle protein degradation and impair muscle regeneration [47, 48].

#### MAPK signalling

The MAPK family, including p38 MAPK and ERK1/2, are involved in regulating various cellular processes, including muscle differentiation, growth, and survival. Dysregulation of MAPK signalling contributes to muscle atrophy by promoting protein degradation and inhibiting protein synthesis [47].

#### Wnt/β-catenin signalling

The Wnt/β-catenin signalling pathway plays a critical role in muscle development, regeneration, and maintenance. Activation of Wnt signalling promotes muscle cell proliferation and differentiation, while inhibition of Wnt signalling contributes to muscle atrophy [49].

In addition to these key signalling pathways, microRNAs (miRNAs), small non-coding RNA molecules, also play a role in regulating muscle mass. miRNAs can modulate gene expression by targeting specific mRNAs, thereby influencing muscle protein synthesis, degradation, and differentiation. Dysregulation of miRNA expression contributes to muscle atrophy in sarcopenia [50].

To prevent sarcopenia, interventions targeting these pathways, such as exercise, nutritional supplementation, and pharmacological approaches, may hold promise for preserving muscle mass and function throughout the lifespan.

### Role of oxidative stress and inflammation in sarcopenia

Oxidative stress and inflammation are key pathological characteristics of skeletal muscle ageing and play a critical role in the development of sarcopenia.

Oxidative stress arises from an imbalance between the production of reactive oxygen species (ROS) and the ability of antioxidant defence mechanisms. With age, ROS production increases while antioxidant capacity declines, leading to oxidative damage of cellular components like proteins, lipids, and DNA [51]. In skeletal muscle, oxidative stress contributes to mitochondrial dysfunction, impairs muscle regeneration, and activates protein degradation pathways [52]. Increased levels of ROS can suppress the phosphorylation of Akt, mTOR and downstream targets p70S6K and 4E-BP1, which are key regulators of protein synthesis [45, 53].

The low-grade inflammation in the elderly, referred to as “inflammaging,” has also been considered a hallmark of ageing and a contributor to sarcopenia [54]. Chronic inflammation exacerbates sarcopenia by increasing the levels of pro-inflammatory cytokines, such as IL-6, IL-1, and TNF-alpha, which leads to increased muscle protein breakdown. This inflammation-induced degradation disrupts muscle reparative processes [55].

The interplay between oxidative stress and inflammation is complex and synergistic. Oxidative stress can trigger the release of pro-inflammatory cytokines, while inflammation can further enhance ROS production. This creates a detrimental cycle that amplifies the effects on skeletal muscle, accelerating muscle atrophy. Considering both mechanisms, therapeutic strategies for sarcopenia may include exercise and potential antioxidant and anti-inflammatory therapies [56, 57].

## Role of protein in sarcopenia

### Anabolic resistance in skeletal muscle

Maintaining muscle protein content is essential to prevent sarcopenia. This is closely linked to the ability to enhance protein anabolism during feeding periods or after exercise, helping to counteract the increased protein breakdown that occurs during fasting, exercise, or both acute and chronic catabolic states. The dietary intake of proteins and/or amino acids (AA) plays a crucial role in stimulating protein synthesis (PS) at both whole-body and skeletal muscle levels. However, this effect is highly dependent on factors such as energy availability, anabolic hormone levels, vascularization, and overall health.

PS could be robustly evaluated in humans using the stable isotope technique. The magnitude of PS stimulation depends on the amount and quality of proteins, which influence the rise in plasma amino acid concentration [58–60]. The activation of PS in humans is dependent on hyperaminoacidemia, notably that of indispensable amino acids(iAA) [61] and, in particular, branched-chain amino acids (BCAA) [61]. Accumulation of evidence obtained in human and animal studies has identified leucine as a major stimulating signal [62]. The process involved the coordinated regulation of several intracellular signalling pathways, notably the IGF-1/PI3K/Akt/mTOR pathway, which leads to the stimulation of protein synthesis. This process is strongly influenced by the timing of nutrient intake, the presence and type of exercise, the concurrent intake of other anabolic nutrients, comorbidities (such as obesity) and the individual’s age. It has been observed that ageing reduces the ability of insulin and amino acids to effectively stimulate muscle PS in both human and animal models [10–12]. The anabolic effect of protein is also impaired with ageing during exercise [63] and obesity was found to further aggravate the effect of ageing in humans [64]Older individuals are often exposed to periods of inactivity due to hospitalisation or chronic diseases. These situations have been shown to accelerate muscle protein loss, as the increased rate of protein breakdown is not adequately compensated by protein synthesis due to anabolic resistance [65, 66]. The role of inflammation in anabolic resistance is also worth considering. Then, the ‘inflammaging’ state has been linked to alterations in muscle protein synthesis in some clinical studies, but the causal link between inflammation and anabolic resistance remains to be confirmed [67, 68]. Another aspect to be investigated to explain the development of anabolic resistance in old individuals is the potential role of satellite cells, as mentioned before [69]. 

###  Dietary protein recommendations for older adults

Food provides proteins for whole-body protein-energy metabolism. Upon ingestion, proteins are broken down by stomach, pancreatic, and intestinal enzymes, releasing AA and small peptides, which are absorbed by the small intestine. Undigested proteins are used by the microbiota in the large intestine. AA enter the bloodstream *via* the portal vein for hepatic metabolism or through the cava vein for peripheral tissue distribution. The primary metabolic pathway for AA is protein synthesis, although certain AA serve specific functions, such as glutathione synthesis, an essential intracellular antioxidant.

The dietary protein requirement, as defined by the Joint WHO/FAO/UNU Expert Consultation, is the minimum intake necessary to balance nitrogen losses and maintain body protein mass under conditions of energy balance and modest physical activity. The FAO recommends an intake for adults of 0.83 g/kg per day for protein [70, 71] close to the US Dietary Reference Intakes, which recommend an intake of 0.8 g/kg per day [72]A meta-analysis confirmed this recommendation, and subsequent studies reaffirmed that protein needs remain consistent regardless of age [73, 74]. However, the recommendations were based on studies using the nitrogen balance method, which has some limitations in the context of older individuals [75]. Considering the development of anabolic resistance and other abnormalities in amino acid metabolism during aging, accumulating evidence supports that increasing protein intake to a range of 1.0–1.2 g per kg per day should be recommended for adults older than 65 years. In fact, while the US RDA for protein remains at 0.8 g/kg/day for all adults, several national and international organisations such as the international study group (PROT-AGE Study Group) of the European Union Geriatric Medicine Society (EUGMS) recommend higher intake for older adults [74](summarised in Table 2). The needs could reach 2.0 g/kg per day in the presence of a chronic disease [76]. Additionally, older adults may need more sulfur-containing amino acids, especially if on long-term medication [77, 78]. While no upper intake limits for amino acids are established due to limited data, protein intake recommendations are continuously updated based on evolving evidence. Despite variations, there is broad agreement on increasing protein intake in older adults, particularly those with sarcopenia or chronic illness, to support muscle health.

**Table 2:** Summary of protein recommendations

Objective	Dose	Organism/Country	References
To support muscle function and prevent related diseases, as sarcopenic situations	1.0 to 1.2 g protein/kg body weight per day for healthy older adults (> 65 years old)	European Society for Clinical Nutrition and Metabolism (ESPEN) and the Society of Sarcopenia, Cachexia and Wasting Disorders (SCWD) The international study group (PROT-AGE Study Group) of the European Union Geriatric Medicine Society (EUGMS)	[79–81]
In cases of severe illness, injury, or protein-energy malnutrition	2 g/kg per day	-	[76]
Over 70 years old	1.07 g/kg for men and 0.94 g/kg for women	Australia and New Zealand	[82]
Over 60 years old	1.0 g/kg	France	[83]
Over 60	0.8 g/kg	US	[72]
Old people	1.2 g/kg, corresponding to 56–81 g/day for men and 46–57 g/day for women	Nordic countries	[84]
-	15–20% of total energy intake	Nordic countries	[84]
	10–35% of total energy intake	US	[85]
Individuals over 60 years old	23% higher need for iAAs	France	[78]
To support muscle protein synthesis	2.5–2.8 g of leucine per meal	US	[85]

### Effect of protein quality on skeletal muscle anabolism

Protein is a broad term encompassing a wide range of structures, each exhibiting distinct properties. Protein quality is an important concept that may provide a small but significant impact on indices of muscle protein anabolism in young and older adults [86]. This issue will be discussed in the following section.

#### Protein quality criteria

Dietary protein quality depends on its iAA composition, as well as its digestibility and absorption by the body. The nutritional value of proteins depends on the bioavailability of their AA and their metabolic utilisation for growth and protein turnover. In 1989, the FAO/WHO established evaluation parameters to assess protein quality [70].

The Protein Digestibility Corrected Amino Acid Score (PDCAAS) is a key indicator that combines true faecal digestibility and the chemical score, reflecting the lowest ratio of an iAA in a protein relative to human needs [70]. Animal-based proteins, such as meat, eggs, and dairy, generally have higher PDCAAS values than plant-based proteins like cereals and legumes. Most plant proteins have PDCAAS scores below 100%, indicating they do not fully meet essential amino acid requirements. Wheat gluten has one of the lowest PDCAAS values (25%), due to low digestibility and deficiencies in essential amino acids. Beyond PDCAAS, the FAO introduced the Digestible Indispensable Amino Acid Score (DIAAS) in 2013, which considers digestible amino acid content and true ileal digestibility rather than faecal digestibility [71]. DIAAS provides a more accurate measure of amino acid absorption, excluding nitrogen contributions from gut microbiota. Both PDCAAS and DIAAS highlight the first limiting amino acid in proteins-sulfur amino acids in legumes and lysine in cereals. Deficiencies in iAA can limit protein synthesis and overall dietary protein quality. In addition to protein digestion extent, digestion speed also impacts protein’s nutritional value for humans [87, 88]. Studies classify proteins as ‘slow’ (e.g., casein) or ‘fast’ (e.g., whey) based on digestion kinetics [88]. Whey proteins are rapidly digested and absorbed, causing a quick but transient rise in blood amino acids, whereas caseins coagulate in gastric acid, leading to slower, prolonged absorption. Soy proteins are digested faster than casein but slower than whey, resulting in a lower postprandial muscle protein synthesis response in healthy adults [89].

#### Effect of whey protein and the consequence of the food transition to more plant-based food

High-quality protein sources are necessary to sustain muscle mass during ageing. Dairy proteins, and specifically whey protein, are very potent for the stimulation of muscle protein synthesis due to their high digestibility and high leucine content. Indeed, in different cell types and tissues, the branched-chain amino acid leucine is a key nutrient regulator of translation initiation and can stimulate muscle protein synthesis [90]. In vulnerable populations such as older adults with sarcopenia, it has been suggested that increasing leucine intake may compensate for the blunted muscle PS to anabolic signals and the age-related decline in muscle mass [91].The beneficial impact of whey proteins (i.e. the “fast” soluble protein fraction of milk proteins) has been demonstrated on muscle protein anabolism in the elderly population and also during exercise training in young subjects [92–94]. In young men, whey protein hydrolysate could induce a higher level of aminoacidemia compared with the free AA mixture providing the same amount of iAA [95], suggesting that a whole-food concept should be considered [95].

The scientific community agrees that increasing the consumption of plant-based foods is essential for transforming both our past and current food systems into more sustainable models. However, plant-based proteins have some limitations in terms of quality compared to animal sources, such as lower digestibility and deficiencies in certain iAA compared to animal proteins. Despite the same leucine intake compared to whey, a dairy/plant protein blend did not restore a positive protein balance in a muscle-wasting situation in minipigs [96]. However, innovations in food processing have solved many issues about plant-based protein digestibility by the production of plant-based protein concentrates, isolates, and hydrolysates. As an example, the weaker postprandial muscle protein synthesis observed in response to the ingestion of hydrolysed wheat protein, compared to whey or casein, could be compensated by increasing the amount of protein ingested [97]. Furthermore, a rodent study suggested that the ingestion of multiple plant protein sources as legumes and cereals, could provide a more balanced amino acid profile and, thus, a great protein retention [98]. Not all plant-based proteins are deficient in iAAs; soy protein, for example, has been shown to stimulate muscle protein synthesis similarly to whey in young men during post-exercise recovery [99]. While dietary strategies based on plant proteins are promising, their effectiveness in stimulating postprandial muscle protein synthesis requires further investigation. Randomised control trials and long-term studies are necessary to evaluate if the threshold and required amounts of proteins or iAAs are similar between plant-based and animal-based proteins.

## Role of polyphenols in sarcopenia

Polyphenols are plant secondary metabolites that contribute to the colour, flavour, and astringency of many fruits, vegetables, and grains. Additionally, plants synthesise them to cope with environmental stressors like UV radiation and microbial attacks. Polyphenols are commonly classified into four main groups; (1) Flavonoids: the most abundant, found in grapes, apples, cocoa, soy, etc.; (2) Phenolic acids: present in black tea, chicory, olives, and walnuts; (3) Lignans: abundant in sesame and olives; (4) Stilbenes: being resveratrol is the most studied; found in grapes and red wine. Although numerous health benefits are linked to polyphenol intake, no official dietary recommendations exist. Intake varies widely by population, ranging from 459 mg/day in Brazil to 1740 mg/day in Poland. This is partly due to the variability in their content across foods, influenced by factors such as climate, cultivation, processing, and storage. Moreover, health effects depend not only on intake but also on bioavailability, which is affected by chemical structure, food matrix, and interactions with gut microbiota.

Polyphenols are renowned for their significant antioxidant and anti-inflammatory properties, among others [100], contributing to various health benefits, including muscle protection against age-related degeneration [100]. Since sarcopenia is strongly associated with oxidative stress and inflammation, polyphenols’ ability to combat oxidative stress and inflammation presents a promising approach to mitigating sarcopenia.

### Antioxidant properties of polyphenols

The antioxidant properties of polyphenols stem from their aromatic rings and hydroxyl groups, which scavenge free radicals to generate resonance-stabilised phenoxyl radicals [101]. Moreover, polyphenols with catechol structures exert antioxidant effects by chelating transition metals like copper and iron, thereby regulating their role in oxygen metabolism [102]. Several studies have shown that diverse polyphenols modulate regulatory enzymes directly implicated in the control of free radical production as cyclooxygenases, lipoxygenases, and specific antioxidant enzymes such as superoxide dismutase (SOD), catalase (CAT), and glutathione peroxidase (GPx) [101]. It has also been shown to enhance endogenous antioxidants such as essential vitamins and increased plasma antioxidant capacity [1]. Derived from all these properties, there is the protection of cellular components, lipids, proteins, and DNA from oxidative damage [1]. Specifically, a mitochondrial function preservation is observed with polyphenols, which could be considered as a key factor in preventing sarcopenia [1].

Myocytes are post-mitotic cells and particularly susceptible to oxidative damage [103]. Their high oxygen consumption increases the production of mitochondria-derived ROS. Moreover, the absence of key antioxidant enzymes, such as peroxiredoxin 6 (Prdx6), increases oxidative stress, leading to telomere shortening and exacerbating muscle atrophy [104–106]. Polyphenols mitigate this damage through three mechanisms. First, they reactivate protein synthesis via the Akt/mTOR pathway [1]. Second, they protect satellite cells from ROS, ensuring continued muscle repair. Third, specific polyphenols like resveratrol activate SIRT1, a key regulator of cellular health and longevity [28].

### Immunomodulation and preservation of muscle integrity

The anti-inflammatory properties of polyphenols have been shown mainly by two mechanisms. The inhibition of pro-inflammatory enzymes and related pathways. These compounds modulate the activity of enzymes such as cyclooxygenase, lipoxygenase, and phospholipase A₂, and interfere with intracellular pathways (NF-κB, MAPK, PI3K/Akt) that control cytokine expression [107]. Polyphenols help to rebalance immune responses by altering gene expression and reducing the release of pro-inflammatory cytokines, which is particularly important in the context of inflammaging and chronic disease.

Polyphenols preserve skeletal muscle function by balancing protein turnover. They suppress inflammatory mediators that promote muscle protein degradation (e.g., via the ubiquitin-proteasome pathway) while enhancing anabolic signalling (Akt/mTOR), polyphenols help maintain muscle mass [13].

### Polyphenols and muscle: evidence from animal and human studies

A population-based analysis of the National Health and Nutrition Examination Survey (NHANES) revealed that moderate dietary flavonoid consumption is linked to a lower risk of frailty in middle-aged and older adults [108]. Numerous systematic reviews and meta-analyses have evaluated the impact of polyphenols on muscle pain and recovery following exercise in healthy adults. Findings suggest that consuming polyphenol-rich foods, juices, and concentrates enhances muscle function recovery and alleviates muscle soreness [108–110].In this line, curcumin supplementation also resulted in an improvement in handgrip strength [111] and muscle fatigue in clinical trials carried out with elderly subjects, with apparent loss of muscle strength [112]. Also, green tea-derived polyphenols, mainly catechins, have been shown to exert promising beneficial effects on sarcopenia. Specifically, green tea polyphenols improved muscle strength and performance in sarcopenic women [113]. Considering these results, polyphenols are important molecules that should be considered when discussing possible strategies against muscle atrophy.

Studies with preclinical models to understand these effectsare scarce and show no clear effectiveness, at least at this moment. Mosoni et al. [109] analysed if supplementation for 6 months with chamomile extract rich in polyphenols (20 g/kg diet) containing apigenin and rutin,, slowed down the loss of lean body mass during ageing in healthy old male rats. Polyphenols did not affect lean body mass or protein synthesis rates measured i*n vivo* (gastrocnemius) and ex-vivo (epitrochlearis) indicating that these polyphenols did not delay sarcopenia. Han et al. [110] studied the effect of another polyphenol-rich extract. In this case, melon peel (*Cucumis melo L*. var. *makuwa*) was administered for 3 weeks after hindlimb-immobilizing 4-week-old male mice for 2 weeks. It was not effective in holding impulses. Soleus and gastrocnemius weights did not vary, nor did the expression of MyoD (soleus), implicated in myoblast proliferation and differentiation.

Regarding human studies, Kwon et al. [111] evaluated the effects of marine oligomeric polyphenols (72 mg/d) (from *Phaeophyceae*) administered for 4 weeks in sarcopenic adults (≥ 65 years) alongside exercise training. The authors compared the results before and after the treatment and with the placebo. Polyphenols increased skeletal muscle mass, indicating that the combination of exercise and polyphenols promotes muscle hypertrophy. However, no difference was observed between groups. Moreover, they increased bone density, without differences with the placebo group. Concerning fitness test measurements, no difference or improvement was observed in grip strength tests, nor in the chair rise test (an indicator of body muscle power). However, they improved agility and dynamic balance (2.4 m up and go test) and static balance (one-leg stand test) after a post-test period. Thus, there are some controversial results between studies which could be partly explained by the diversity of the structures considered, the limited number of studies developed and the different study approaches run in each one.

Resveratrol remains the most extensively investigated polyphenol for sarcopenia intervention. While evidence supports its therapeutic potential, outcomes are highly sensitive to dosage, treatment duration, and lifestyle co-interventions. In this regard, data on muscle mass preservation are heterogeneous. High-dose supplementation appears necessary to elicit structural changes in specific models. In obese mice, 200 mg/kg of resveratrol increased skeletal muscle weight, whereas lower doses (100 mg/kg) primarily targeted fat mass. Similarly, in senescence-accelerated mice, a dosage of 150 mg/kg combined with exercise significantly reduced cell apoptosis and preserved fiber space, outperforming either intervention alone [112]Conversely, lower-dose or chronic regimens show limited efficacy. Jackson et al. observed no protective effect on muscle weight, mitochondrial content, or PGC1α levels in mice fed a 0.05% resveratrol diet for 10 months [113].

In another study, 25-week-old male rats were fed with a standard diet supplemented or not with resveratrol (150 mg/kg BW/day) for 6 weeks. Resveratrol reduced body mass gain but had no gastrocnemius muscle indexes of the hindlimbs, nor on absolute grip strengths. However, it increased relative grip strength [114].

In a different study, 28-week-old male mice were fed with a control diet containing or not resveratrol (400 mg/kg diet). Resveratrol mitigated ageing-related motor dysfunction and skeletal muscle atrophy while restoring muscle protein acetylation and autophagy, indicating a suppression of sarcopenia [114].

In another experiment, 25-month-old male rats were assigned to the following groups: control, exercise-treated, resveratrol-treated (150 mg/kg BW/day) for 6 weeks, and exercise + resveratrol-treated. Exercise reduced body weight and increased relative grip strength similarly in all rats, as well as Edstrom’s Sarcopenia Index (gastrocnemius muscle weight (mg)/BW (g)). The sarcomere length, I-band length and H-zone length were higher in the control group, without differences among the other groups. Moreover, rats fed resveratrol showed a higher perimeter of gastrocnemius fibres compared to the control group,, without differences among groups in Feret’s diameter. Thus, resveratrol and training improve skeletal muscle mass and muscle function [115].

Caloric restriction helps to slow down the age-related decline in muscle fibres by enhancing mitochondrial function and decreasing apoptosis. Given that, a study [116]assessed the efficacy of caloric restriction (20% of energy), resveratrol (50 mg/kg BW/day), or the combination of both in 27-month-old male rats during 6 weeks [116]. Caloric restriction reduced BW, soleus and plantaris weights, and gastrocnemius weight decreased when combining caloric restriction and resveratrol. When muscle weights were adjusted for body weight, it was observed an increase in gastrocnemius in rats under caloric restriction (without resveratrol), in plantaris by the combination of both treatments and in soleus in all animals under caloric restriction. No changes in mitochondrial function were observed. To our knowledge, no studies have been carried out focused on the actions of resveratrol in aged female animals.

Moving on to clinical trials with old people, it has beeno demonstrated that resveratrol enhances muscle fatigue resistance during exercise [117]. Particularly, a study from 2017 [118]was carried out in 65–80 years-old healthy men and women who were randomly assigned to a group that received resveratrol (500 mg/d) (or not) together with exercise (resistance and aerobic training) for 12 weeks [118]. Unfortunately, no differences between groups were observed in weight, lean mass or body fat. Regarding cardiovascular adaptations, there were no significant differences in absolute VO_2_ max between groups, but it was improved by exercise when combined with resveratrol (compared pre-and post-treatment). Muscle mitochondrial density and anti-apoptotic proteins were only increased in resveratrol-treated subjects. Concerning muscle strength and fatigue, only the combination of resveratrol and training increased knee extensor strength (peak torque, average peak torque and muscle power). In the case of power, the differences were significant between the two groups. These improvements could not be attributed to differences in muscle fibre type. However, resveratrol increased nuclear and satellite cell numbers (useful for muscle regeneration and hypertrophy) of exercised muscles. These results suggest that resveratrol in combination with exercise could mitigate or reverse sarcopenia in older adults.

To sum up, despite these promising functional data, outcomes are highly sensitive to dosage, treatment duration, comorbidities, and age. Moreover, the literature contains a critical demographic gap: the vast majority of animal studies utilise male models. Future research must address this disparity to determine if these findings translate to ageing female populations.

## Synergistic effects of protein and polyphenols

As previously described, the effects of protein intake and polyphenols have gained attention due to their ability to modulate key molecular pathways involved in muscle health [119].

However, all these nutritional strategies (polyphenols and proteins) have been separately considered, whereas little is known about the combined effects of nutraceutical supplementation with both polyphenols and proteins to further enhance the benefits of the nutritional intervention in the context of muscle loss and other pathologies related to sarcopenia. The combined intake of protein and polyphenols could offer significant benefits [120], improving polyphenol bioavailability and modulating the food protein structure leading to a significant reduction in its allergenicity and improving its digestibility [121–125]. In fact, binding polyphenols with proteins protects them from enzymatic and oxidative degradation, improves their stability during digestion, and consequently enhances their intestinal absorption [125, 126]. As an example, (-)-epigallocatechin gallate (EGCG) is commonly utilised for protein modification to enhance its functional characteristics [121].

Similarly, and as mentioned, interactions between polyphenols and proteins can significantly enhance polyphenol bioavailability, targeted delivery and biological activity [125], which could enhance their actions on the muscle.

However, it is important to underline that traditional methods for preparing protein–polyphenol conjugates have serious drawbacks, such as low efficiency and long reaction times, which limit the large-scale commercial production and application of these functional components [127, 128]. Therefore, many studies have explored more efficient methods that can potentially replace the traditional techniques, such as ultrasounds methods with a very positive perspective according to the study from Yan et al. [127]. However, more research is needed to fully understand the physiological effects and mechanisms of these interactions to refine engineering design of polyphenols.

Returning to the potential use of polyphenols and proteins as a synergistic nutritional mechanism against sarcopenia, only a recent study has observed that enhancing protein and polyphenol intake, through the supplementation of fermented black soybean koji product (BSKP) (polyphenol-rich plant-based proteins) attenuated age-related sarcopenia by inducing antioxidant enzymes and short chain fatty acids production via gut microbiota regulation in Taiwan´s community-dwelling elderly [129]. This study reported that BSKP supplementation significantly increased the appendicular skeletal muscle mass index and reduced LDL levels, which was one of the biomarkers for early diagnosis of sarcopenia [130]. These effects were associated with gut microbiota remodelling, including enhanced antioxidant enzyme activity and elevated faecal butyrate levels [129]. Notably, reduced faecal butyrate levels have been linked to lower skeletal muscle mass in the elderly [131]. These anabolic effects support muscle mass preservation and may serve as a strategy to slow sarcopenia progression in elderly individuals. However, its extrapolation into a general population is quite difficult due to the origin of the individuals that participated in the study (Taiwan).

Looking into the underlying mechanisms of such effects, Pavis et al. demonstrated that the combination of protein and polyphenols enhanced myofibrillar protein synthesis and early muscle function improvements in athletes. These findings demonstrate for the first time that daily ingestion of a protein-polyphenol beverage, known to suppress muscle damage, increases myofibrillar protein synthesis within 48 h of initial resistance training by potentially modulating molecular pathways related to muscle protein metabolism, thereby accelerating early muscle function improvements and adaptation [132].

However, other studies suggested that while polyphenols improved oxidative stress and inflammation, the combination of proteins and polyphenols did not influence muscle protein metabolism in healthy rats. This lack of effect on muscle protein metabolism may be due to the rats’ exceptionally good oxidative and inflammatory status, which, despite slight deterioration over six months, remained within a healthy range and, therefore, additional polyphenol supplementation had no impact on lean body mass or muscle protein synthesis. However, such supplementation could still be beneficial in conditions of low-grade inflammation [109]. Thus, further in vivo and in vitro studies are needed to better understand the effects of combined protein and polyphenol consumption on sarcopenia, as well as to determine their potential mechanisms and therapeutic potential in humans.

**Fig. 1:**
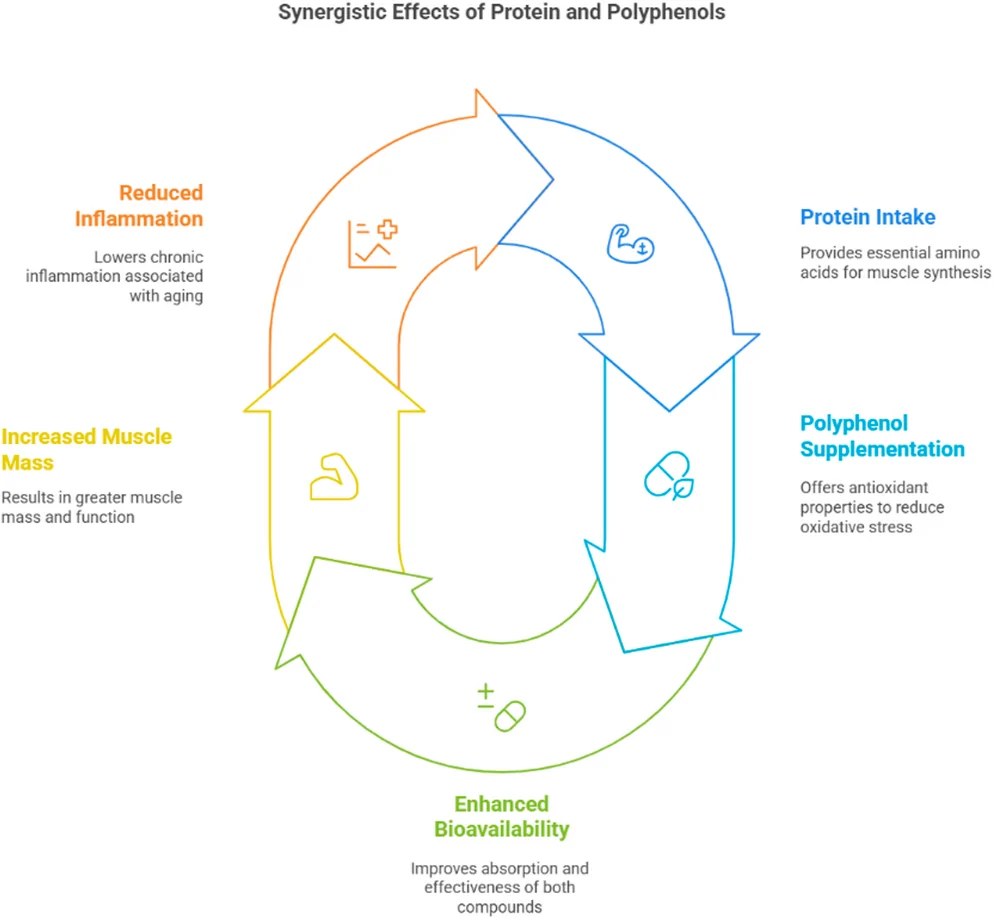
Synergetic effects of protein and polyphenols in sarcopenia condition

To sum up, more research is needed to explore the combined effects of proteins and polyphenols supplementation on muscle health, focusing on both food technology innovations to design enriched products and also more detailed in vivo and clinical studies to better understand the mechanisms and potential therapeutic benefits for sarcopenia. (Figure 1.)

## Other strategies for sarcopenia management

### Other nutritional strategies

Integrated nutritional interventions play a crucial role in managing sarcopenia. Previously, we have considered adjusted protein intakes, with or without polyphenol intake. However, it must be also considered an adequate intake of other essential micronutrients, such as vitamins and minerals along with the addition of specific supplements, which support muscle health and growth [133],. Vitamin D plays a crucial role in muscle function and may contribute to improved physical activity levels and muscle health in older adults. Adequate vitamin D levels are linked to better muscle function and physical activity [73], while its deficiency may impair type II muscle fibres [74]. Thus, sarcopenia is strongly linked to vitamin D deficiency [134]. In this sense, Gkekas et al. reported that supplementation with vitamin D combined with protein confers a beneficial effect on muscle strength in patients with sarcopenia [135]. In addition, supplementation with vitamin D has shown benefits, including increased type II fibres and improved muscle quality, especially when combined with resistance training [75], though further research is needed.

Antioxidants may play a role in managing sarcopenia by reducing oxidative stress, a key factor in muscle decline. Vitamins C and E, known for their ability to scavenge reactive oxygen species (ROS) and enhance cellular antioxidant capacity, may help preserve muscle mass and function. These antioxidant vitamins have been demonstrated to have potential effects in vitro, although there is not enough convincing evidence about their effects on humans [136].

Recent research has also analysed the potential benefits of polyunsaturated fatty acids in promoting muscle health and counteracting the progression of sarcopenia. Adequate intake of n-3 polyunsaturated fatty acids has been linked to better total-body and appendicular skeletal muscle mass, while a higher n-6/n-3 ratio appears to reduce muscle mass [70]. Many studies have also demonstrated that oral intake of omega-3 PUFA has a great impact on muscle mass and function in older adults [137–139].

Nutritional interventions for sarcopenic patients have incorporated probiotics, prebiotics, and symbiotics, which have been shown to mitigate muscle mass loss while enhancing grip strength and gait speed [140, 141]. These studies suggest that these bioactive compounds exert their beneficial effects on muscle mass loss by the reduction of systemic chronic low-grade inflammation associated with sarcopenia. Additionally, a food product containing omega-3 fatty acids, leucine, and the probiotic LPPS23 seems to be an effective strategy to combat sarcopenia and its defining parameters in older adults [69].

In conclusion, nutrition is crucial in managing sarcopenia, and further research is needed to optimise nutrient combinations and fully understand their impact on muscle health in ageing populations.

### Role of exercise in combination with nutrition

Supplements have emerged as a promising nutritional strategy for managing sarcopenia, particularly when combined with exercise. Indeed, creatine supplementation has been shown to increase lean tissue mass and muscle strength in ageing adults. Compared with resistance training alone, creatine supplementation improves muscle strength, with greater gains in lean tissue mass resulting from post-exercise creatine supplementation [76, 77].

Different clinical trials evaluated the effects of supplementation with amino acids or proteins in combination with exercise training in healthy older adults in sarcopenic adults. Kim et al. proved that exercise in combination with amino acid supplementation produced significant effects on enhancing muscle strength, muscle mass and walking speed in sarcopenic women [142]. Also, Bonnefoy et al. indicated similar effects produced by the combination of a nutritional supplement rich in protein and micronutrients, combined with physical exercise in the frail elderly population [143]. Apart from protein supplementation, different clinical studies have included other supplements in combination with exercise to improve sarcopenia. In this sense, training in combination with supplementation with fish-oil, rich in n–3 (omega-3) polyunsaturated fatty acids (PUFAs) resulted in a greater improvement in muscle strength and functional capacity than training by itself in elderly women [144]. Other studies included the combination of exercise with supplementation with vitamin D, resulting in an improvement of physical performance in the elderly population [145]. Similarly, another clinical trial demonstrated enhanced benefits of exercise training when combined with creatine supplementation [146].

The observed enhancement of exercise benefits in sarcopenia when paired with dietary supplementation, according to results obtained in different clinical trials, should be considered as a potential strategy for preventing and managing sarcopenia. However, it is still difficult to establish clear recommendations, and existing evidence is based on populations who differ in age, frailty, and nutritional status and in consequence, some results remain inconclusive.

## Emerging trends and future research directions

Current sarcopenia therapies primarily focus on nutrition and physical exercise, but emerging strategies are exploring molecular pathways and novel biomarkers to improve outcomes. In this sense, myostatin inhibition, through monoclonal antibodies targeting this negative regulator of muscle growth, has demonstrated promise in preclinical models for increasing muscle mass and strength [147, 148], with ongoing clinical trials assessing safety and efficacy in older adults. Mitochondrial therapeutics are also under investigation, as mitochondrial dysfunction, linked to oxidative stress and impaired mitophagy, contributes to sarcopenia. Thus, approaches including mitochondria-targeted antioxidants like MitoQ and agents that enhance mitophagy are being researched [149, 150]. Another promising area is the gut-muscle axis, where modulation of gut microbiota using probiotics, prebiotics, or polyphenol-rich diets may reduce systemic inflammation and promote butyrate production, potentially mitigating muscle loss [151–153]. In addition, gene editing techniques such as CRISPR/Cas9 and stem cell therapies are being explored to regenerate muscle tissue by targeting atrophy-related genes or activating satellite cells [154, 155]. Hydration and electrolyte balance are increasingly recognised as key factors in maintaining muscle function, particularly in elderly individuals at risk of dehydration [150, 156]. Finally, personalised medicine approaches are gaining traction, emphasising the identification of early biomarkers—such as myostatin levels or gut microbiota profiles—to enable individualised, targeted interventions. Combining traditional therapies with novel strategies, including anti-inflammatories and mitochondrial enhancers, may offer synergistic benefits in preventing and treating sarcopenia [149, 157].

## Conclusion

**Fig. 2:**
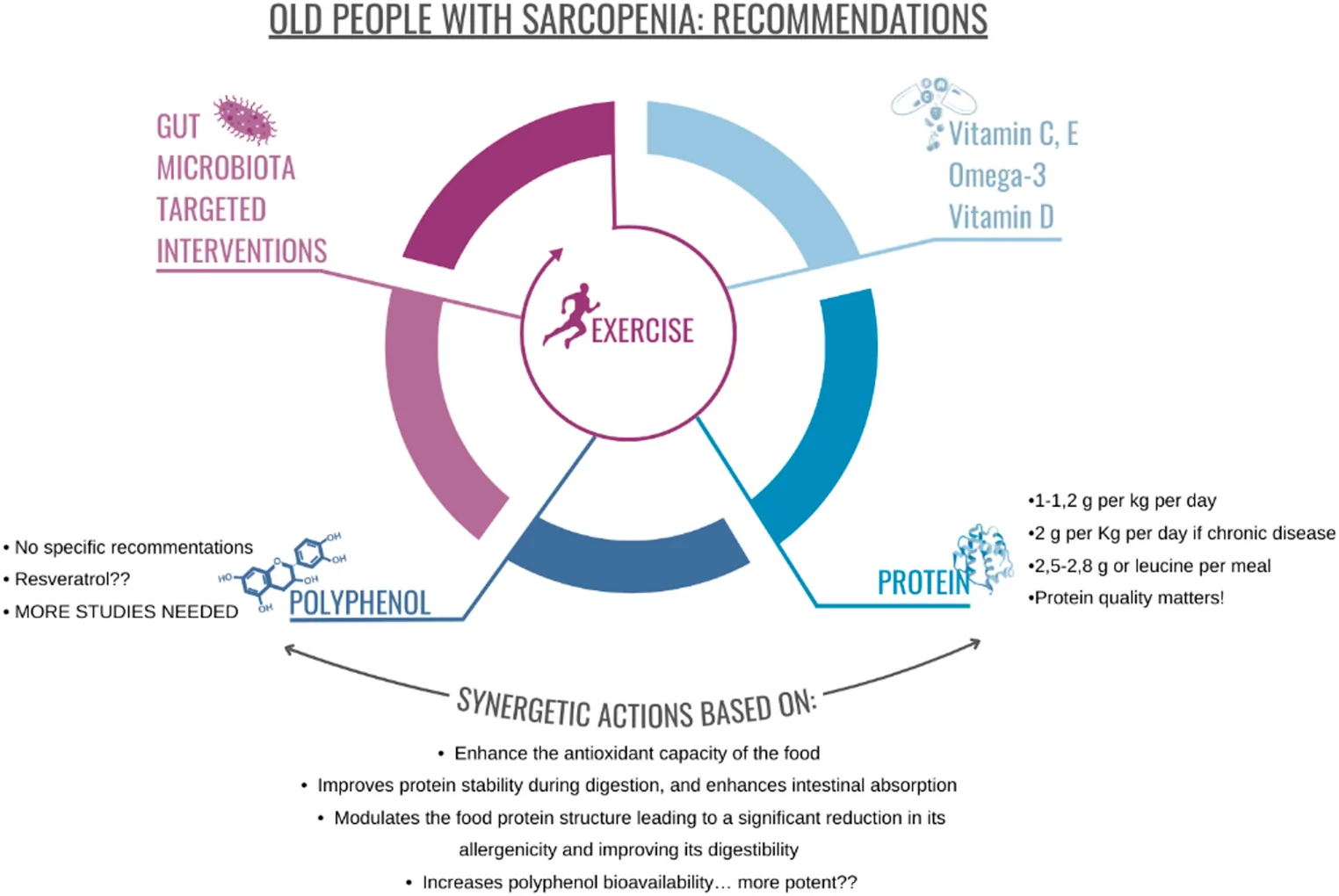
Take-home key recommendations for sarcopenia

Sarcopenia remains a complex and multifactorial condition, with its pathophysiology involving numerous interconnected biological processes. This complexity presents both challenges and opportunities for the development of targeted and effective interventions. Current evidence increasingly supports optimising protein intake, with particular attention to high-quality protein sources and amino acids such as leucine, to counteract anabolic resistance and preserve muscle mass in older adults. Emerging data on plant-based proteins and bioactive compounds, including polyphenols, highlight promising avenues but remain insufficient to support specific clinical recommendations. Importantly, combining tailored nutritional strategies with structured physical activity has shown additive benefits, underscoring the need for integrative, multimodal approaches (Fig. 2). However, significant heterogeneity across study designs, populations, and intervention protocols continues to limit the formulation of standardised guidelines.

This review is inherently shaped by the uneven distribution of evidence across domains, with robust human data in some areas and a reliance on preclinical studies in others. Continued high-quality research, particularly in diverse human populations, is therefore essential to refine and personalise strategies for the prevention and management of sarcopenia.

## Data Availability

The authors declare that all data were generatedin-house, although during the preparation of this work, the authors usedChatGPT (OpenAI) in order to enhance the readability and language of the manuscript. The authors reviewed and edited the content as needed and take full responsibility for the content of the publication.
